# Assessment of worries and attitudes towards the COVID-19 pandemic and the vaccine among Tunisian elderly

**DOI:** 10.1192/j.eurpsy.2022.1275

**Published:** 2022-09-01

**Authors:** M. Moalla, R. Lansari, R. Saida, M. Zrelli, A. Larnaout, W. Melki

**Affiliations:** Razi Hospital, Psychiatry D, Manouba, Tunisia

**Keywords:** attitudes, Covid-19, worries, Elderly

## Abstract

**Introduction:**

The COVID-19 pandemic poses a threat particularly to the elderly. Although the current vaccination strategy is recognized as an adequate measure to reduce mortality, it still raises concerns about its efficacy and safety.

**Objectives:**

Assessment of worries and attitudes among Tunisian elderly towards the pandemic.

**Methods:**

A descriptive cross-sectional study on a sample of 50 consultants in a geriatric service, aged between 65 years and over. A questionnaire was formulated based on the recommendations of WHO and INEAS.

**Results:**

The average age of our population is 74.6 years. The participants were mainly female, retired (76%) and with low educational attainment. Most of the elderly reported that they respected the wearing of the mask in public (90%) and washing their hands regularly (92%). Social distancing was respected by only 44% of the participants. Concerning the vaccine registration, we noted that 48% of the subjects expressed their willingness to register on the Evax.tn platform. On the other hand, 15 people expressed their refusal to receive the anti-Covid vaccine. We noted that only 22% had a dose of the Covid-19 vaccine. Only 4% of the respondents did not have concerns about new variants of the virus. About half (52%) of the subjects expressed significant concern about an increased risk of virulence and mortality due to the new variant. Vaccination was considered ineffective by most of the participants (70%).

**Conclusions:**

Addressing worries about vaccine would be an important step to accept it among Tunisian elderly. Adequate information strategy is essential to change attitudes during the pandemic.

**Disclosure:**

No significant relationships.

